# Serological and molecular analysis of feline vector-borne anaplasmosis and ehrlichiosis using species-specific peptides and PCR

**DOI:** 10.1186/s13071-015-0929-8

**Published:** 2015-06-12

**Authors:** Barbara C. Hegarty, Barbara A. Qurollo, Brittany Thomas, Karen Park, Ramaswamy Chandrashekar, Melissa J. Beall, Brendon Thatcher, Edward B. Breitschwerdt

**Affiliations:** Intracellular Pathogens Research Laboratory, College of Veterinary Medicine, North Carolina State University (NCSU), 1060 William Moore Dr., Raleigh, NC 27607 USA; IDEXX Laboratories, Inc, Westbrook, ME USA

**Keywords:** *Anaplasma*, *Borrelia*, *Ehrlichia*, Cats, Vector-borne pathogens

## Abstract

**Background:**

With the exception of *Bartonella* spp. or *Cytauxzoon felis*, feline vector-borne pathogens (FVBP) have been less frequently studied in North America and are generally under-appreciated as a clinical entity in cats, as compared to dogs or people. This study investigated selected FVBP seroreactivity and PCR prevalence in cats using archived samples.

**Methods:**

Feline blood samples submitted to the Vector Borne Diseases Diagnostic Laboratory (VBDDL) at North Carolina State University College of Veterinary Medicine (NCSU-CVM) between 2008 and 2013 were tested using serological assays and PCR. An experimental SNAP® Multi-Analyte Assay (SNAP® M-A) (IDEXX Laboratories, Inc. Westbrook, Maine, USA) was used to screen all sera for antibodies to *Anaplasma* and *Ehrlichia* genus peptides and *A.phagocytophilum*, *A.platys, B.burgdorferi, E.canis, E.chaffeensis*, and *E.ewingii* species-specific peptides. PCR assays were used to amplify *Anaplasma* or *Ehrlichia* DNA from extracted ethylenediaminetetraacetic acid (EDTA)-anti-coagulated blood samples. Amplicons were sequenced to identify species.

**Results:**

Overall, 7.8 % (56/715) of cats were FVBP seroreactive and 3.2 % (13/406) contained *Anaplasma* or *Ehrlichia* DNA. Serologically, *B.burgdorferi* (5.5 %) was the most prevalent FVBP followed by *A.phagocytophilum* (1.8 %). *Ehrlichia* spp. antibodies were found in 0.14 % (12/715) of cats with species-specific seroreactivity to *E.canis* (*n* = 5), *E.ewingii* (*n* = 2) and *E.chaffeensis* (*n* = 1). Of seropositive cats, 16 % (9/56) were exposed to more than one FVBP, all of which were exposed to *B.burgdorferi* and either *A.phagocytophilum* (*n* = 7) or *E.ewingii* (*n* = 2). Based upon PCR and DNA sequencing, 4, 3, 3, 2, and 1 cat were infected with *A.phagocytophilum, A.platys, E. ewingii, E. chaffeensis* and *E.canis*, respectively.

**Conclusions:**

Cats are exposed to and can be infected with vector-borne pathogens that commonly infect dogs and humans. To our knowledge, this study provides the first evidence for *E.chaffeensis* and *E.ewingii* infection in naturally-exposed cats in North America. Results from this study support the need for regional, serological and molecular FVBP prevalence studies, the need to further optimize serodiagnostic and PCR testing for cats, and the need for prospective studies to better characterize clinicopathological disease manifestations in cats infected with FVBP.

## Background

In North America, fleas, mosquitoes and ticks are considered the most important vectors for transmission of a spectrum of infectious agents that can induce disease in mammalian species, including dogs and humans; however, with the exceptions of *Dirofilaria immitis* (mosquito-borne feline heartworm disease) [1], *Cytauxzoon felis* (tick-borne feline cytauxzoonosis) [2] and *Bartonella henselae* (flea or tick-borne feline bartonellosis) [1], other known canine and human tick-borne pathogens have not been detected in, or have been minimally studied, in cats in the US and throughout much of the world when compared to dogs or humans. *Anaplasma*, *Borrelia* and *Ehrlichia* species infect cows, sheep, dogs, horses and human beings; however, the role of these pathogens as a cause of disease in cats remains incompletely defined [3]. As compared to dogs and humans, the smaller number of FVBP studies may be due to the lack of standardized serological tests, either ELISA or IFA, as are used routinely in canine veterinary practices. Also, veterinarians’ perception of the risk of FVBD may be a factor because there have been fewer research studies defining the regional serological or PCR prevalence of anaplasmosis, ehrlichiosis, and Lyme disease in cats. This factor is undergoing rapid change as researchers around the world have begun to investigate FVBP prevalence among various cat populations (feral, healthy, sick, etc.). Serological, and to a lesser extent molecular-based, FVBP studies have been reported from the US [4–6], Brazil [7–9], France [10], Portugal [11], Italy [12] Spain [13, 14], Sweden [15], Kenya [16], and the Far East [17, 18]. In parts of Spain and Italy, stray and domestic cat *A.phagocytophilum* seroprevalence rates ranged from 2–8 % [7, 19, 20]. In the US, *A.phagocytophilum* seroprevalences range from 4.3 % [6] in southeastern US to as high as 38 % in northeastern US endemic regions [5]. *E.canis* seroprevalence rates have ranged from 6–18 % in Europe [7, 13, 14, 19, 20]. In the Western hemisphere fewer *Ehrlichia* spp. seroprevalence studies have been performed; however, *E.canis* seroprevalence was 5.5 % amongst 200 domestic cats in Brazil [8]. *B.burgdorferi* seroprevalence rates as high as 47 % were found in cats from areas endemic for Lyme in the US [5]. Currently, veterinary diagnostic serological assays rely upon *A.phagocytophilum, B.burgdorferi* and *E.canis* antigens and assays that were originally validated for testing dogs and in most instances have not been optimized for testing cats.

Molecular-based evidence, such as PCR, indicates that cats can potentially be infected with *A.phagocytophilum* [4, 12, 15]*, A.platys* [21, 22] and *E.canis* [9, 11, 23–25]. In Sweden, Bjoersdorff *et al.* was first to report PCR amplification of *A.phagocytophilum* from a cat [15]. The DNA sequence in a 14-month-old shorthaired cat with lethargy and fever was 100 % identical to dog and horse *A.phagocytophilum* strains from the same region. Subsequently, Lappin *et al.* confirmed infection with *A.phagocytophilum* in 5 young clinically-ill cats from the northeastern US by PCR amplification and DNA sequencing [4]. To date, *A.platys* infections, with PCR amplification of the pathogen, have been reported in only two thrombocytopenic cats [21, 22]. Other clinical manifestations included anorexia and platelet inclusion bodies in a cat from Brazil [21] and chronic hyperglobulinemia in a cat from North Carolina that was also PCR positive for *Mycoplasma hemominutum, Bartonella henselae* and *Bartonella koehlerae* [22]. Using PCR, *Ehrlichia* species DNA has been amplified from cats located in Italy [12], France [10, 23] and the Americas [8, 9, 23–25]. DNA evidence of possible *E.canis* (98 % identity) and *E.chaffeensis* (97 % identity) infections was reported by Braga Mdo *et al.* in cats from Brazil [8]. Positive *E.canis* PCR results were also reported for 11 thrombocytopenic and lymphopenic Brazilian cats [9, 25]. Based upon PCR amplification and DNA sequencing of the *Ehrlichia 16S* rDNA gene, our research group described *E.canis* infection (100 % identical to *E.canis* DNA obtained from dog isolates) in cats from France [23] and in 3 young, sick cats from the southeastern United States or eastern Canada [24].

IDEXX Laboratories, Inc. (Westbrook, Maine, USA) developed a qualitative enzyme-linked immunosorbent assay (ELISA) test, SNAP® M-A, using *Anaplasma* genus EENZ1 and *Ehrlichia* genus p30/p30-1 peptides to broadly detect *Anaplasma* spp. and *Ehrlichia* spp. antibodies in conjunction with *A.phagocytophilum* p44 Aph, *A.platys* p44 Apl*, E.canis* p16*, E.chaffeensis* VLPT*, E.ewingii* p28, and *B.burgdorferi* C6 species-specific peptides as a research tool to characterize regional trends in seroprevalence to specific vector-borne pathogens in dogs [26, 27]. Although developed as a canine assay, the assay does not use a host species-specific conjugate, and can therefore be used on a research basis to screen mammalian species other than dogs. An earlier ELISA based assay, the SNAP® 3Dx®, was used in a serosurvey of cats naturally exposed to *B.burgdorferi* [28] and the SNAP®4Dx® has been utilized to test horses for borreliosis [29] and ehrlichiosis [30, 31]. Recently, Qurollo *et al.* reported seroprevalence data using SNAP® M-A in over 6500 archived canine serum samples from the US, Canada and the Caribbean. Overall and regional seroprevalence and co-seroprevalence (exposure to more than one pathogen) was determined, as well as seroprevalence trends between 2008 and 2010 [26]. Although currently SNAP® M-A is not available commercially, the use of a broad range of genus and species-specific immunodominant peptides in diagnostic tests would allow veterinarians to determine *Anaplasma* or *Ehrlichia* species exposures in dogs and cats in their region. This information could benefit both clinical decision making, as well as human and veterinary zoonotic disease education [32–34].

The purpose of this study was to evaluate a wide range of FVBPs in cats suspected of exposure to a vector-borne pathogen. Specifically, we determined the seroprevalence of *Anaplasma* spp*., B.burgdorferi* and *Ehrlichia* spp. and the presence of *Anaplasma* or *Ehrlichia* sp. DNA, as determined by PCR amplification and DNA sequencing.

## Methods

All samples were de-identified, so no ethical approval necessary.

### Clinical accessions and sample availability

Feline clinical accessions (*n* = 858) submitted to the NCSU-CVM-VBDDL between January 1, 2008, and December 31, 2013, for FVBP testing originated from veterinary hospitals located in the United States (*n* = 827), Canada (*n* = 28) and the Caribbean (*n* = 3). Sample submission information included date of collection, date received, and the cat owner or veterinary practice address while individual identifications were not revealed. Of these 858 cats, 715 sera were available for testing using the SNAP® M-A kit for *Anaplasma, Borrelia, and Ehrlichia* antibodies and EDTA-anti-coagulated whole blood was available from 406 cats for PCR testing. Prior to the study, *Anaplasma* and *Ehrlichia* diagnostic PCR results were known for 163 of these 406 cats. Stored, frozen (−80 °C) blood was retrievable for additional PCR testing for 331/406 diagnostic accessions. When adequate serum volumes were available, a subset of 59 SNAP® M-A seropositive and 4 PCR positive samples were tested by SNAP®4Dx®Plus assay (IDEXX Laboratories, Inc. Westbrook, Maine, USA) and by indirect fluorescent antibody (IFA) assays using whole cell antigens. Medical record data was obtainable for 7 PCR positive cats, 4 of which were referrals to the NC State Veterinary Hospital and 3 were from other veterinary hospitals or universities. Medical records were reviewed for the signalment, history and physical examination findings, complete blood count (CBC), serum biochemistry profile abnormalities and for additional diagnostic testing that were performed, including abdominal ultrasound, radiographs, or coagulation profiles.

#### Serological assays

Cat serum specimens (*n* = 715) were retrospectively tested by SNAP® M-A for *A.phagocytophilum*, *A.platys*, *B.burgdorferi, E.canis*, *E.chaffeensis*, and *E.ewingii* antibodies. This kit uses a reversible chromatographic flow of sample and automatic, sequential flow of wash solution and enzyme substrate. Archived cat serum stored at − 80 °C was thawed to room temperature prior to mixing 4 drops of serum with 4–5 drops of SNAP® M-A conjugate. The mixture was allowed to move across a flow matrix where peptide-specific antibody could bind to peptide-horse radish peroxidase conjugate before color reactant release. Color development, indicating a positive reaction, was read after 15 min per manufacturer’s instructions (IDEXX Laboratories, Inc, Westbrook, Maine, USA).

#### PCR testing

DNA from 200 μL of feline EDTA-anti-coagulated whole blood was extracted with a QIAsymphony™ robotic extractor using MagAttract® DNA Mini M48 kit (Qiagen, USA cat: 953336). DNA quality was assayed by quantitative PCR (qPCR) amplification of the glyceraldehyde 3-phosphate dehydrogenase gene on feline genomic DNA. A conventional PCR assay targeting a conserved region of the *Anaplasma* and *Ehrlichia 16S* rRNA gene [35] and two qPCR assays targeting the *Anaplasma* genus *Tr-1* and *Ehrlichia* genus *sodB* [36] genes were performed on stored, frozen blood samples (*n* = 331). Species were identified by amplicon sequencing (Genewiz, Research Triangle Park, NC, USA) and additional, species-specific qPCRs were used in an attempt to confirm positive samples. The *Anaplasma* genus *Tr-1* assay and species-specific assays (*A.phagocytophilum p44, A.platys p44, E.chaffeensis nadA, and E.ewingii sodB*) used in this study have not been previously reported; primer sequence and amplicon size for each of these qPCRs are listed in Table 1. The reactions were performed in a CFX96™ Real-Time Detection System combined with C1000™ Thermal Cycler (Bio-Rad, USA) under the following conditions: 25 μl final volume reaction containing 12.5 μl of SYBR®Green Supermix, 0.2 μM of the *Tr-1* primers and 0.3 μM of each species-specific primer (Sigma-Aldrich, St Louis, MO, USA), 7 μl of filter-sterilized, molecular-grade water and 5 μl of DNA template. Thermocycler conditions consisted of a single cycle at 98 °C for 2 min, followed by 40 cycles of denaturation at 98 °C for 15 s, annealing at 57 °C (*Tr-1*) or 67 °C (all species-specific assays) for 15 s, and extension at 72 °C for 15 s. Melting temperature measurements were made between 65–88 °C at 0.5 s intervals.

**Table 1:** Primer targets, sequences and amplicon base pair size for previously unreported quantitative PCR assays used in this study. F: Forward primer; R: reverse primer

Target	Primers	Amplicon (bp)
*Anaplasma -tr-1*	F- 5′ ATGTTTATGACTTCTCAAGCAC-3′ R- 5′ CCC TTT TCG TAT TTT TGT AC-3′	200
*A. phagocytophilum -p44*	F- 5′ TGGTGGTGCGGGATATTTCTATGTTG-3′ R- 5′ CCCAATCCGAGGATCAGGTGTG-3′	179
*A. platys -p44*	F- 5′ GCT AAG TGG AGC GGT GGC GAT GAC AG-3′ R- 5′ GCCGCAGTTTCCCCGGTACT-3′	179
*E. chaffeensis -nadA*	F- 5′ CGCAAAAGATGTAATTCTTTGGGAATC-3′ R- 5′ CACCTCAAAATCAGAATTCATCGAAGG-3′	117
*E. ewingii -sodB*	F- 5′ GCTGGAATAGGTCATTTTGGTAGTGGA-3′ R- 5′ GTTCCCATACATCCATAGCAAGCAAC-3′	147

#### Comparison of serological assays

Fifty-nine stored, frozen sera from SNAP® M-A seropositive and/or PCR positive cats were retested using the SNAP®4Dx®Plus assay (IDEXX Laboratories, Inc. Westbrook, Maine, USA) according to manufacturer’s directions. SNAP® M-A and PCR positive cats were also tested by IFA using *A.phagocytophilum* (ProtaTek International Inc. St. Paul, MN, USA), *E.canis* (strain NC Jake), *E.chaffeensis* (strain *E.chaffeensis* Ark) and *B.burgdorferi* (MBL Bion, Des Plaines, IL, USA) antigen slides as appropriate for the seroreactivity detected by SNAP® M-A. For IFA testing, serum samples were diluted two fold from 1:16 to 1:512 in phosphate buffered saline (PBS) solution containing 1 % normal goat serum, 0.05 % Tween-20 and 0.5 % powdered nonfat dry milk (BioRad, Hercules, CA, USA). Antibody responses were detected by IFA with fluorescein conjugated goat anti-cat IgG (Thermo Fisher Scientifics, Waltham, MA, USA) [37]. Seropositive samples were defined as having endpoint titers ≥ 1:64 based upon laboratory criteria used by the VBDDL.

## Results

### Seroreactivity

Of the 715 cat serum samples tested with SNAP® M-A, the overall FVBP seroprevalence (Table 2) was 7.8 % (56/715) with *B.burgdorferi* the most seroprevalent at 5.5 % (39/715). By SNAP® M-A testing, seroreactivity to an *Anaplasma* or *Ehrlichia* spp. peptide was detected in 2.9 % (21/715) of cat serum accessions tested. Based upon the 3 *Anaplasma* spp. analytes, 1.8 % of cats (13/715) were exposed to an *Anaplasma* spp., of which 12 were *A.phagocytophilum* seroreactive and one cat was seroreactive with only the genus analyte. No cat was SNAP® M-A *A.platys* seroreactive. Using SNAP® M-A, *Ehrlichia* genus seroreactivity was found in 1.7 % (12/715) of cats. Based upon reactivity to a species-specific peptide, 5 of the 12 cats were seroreactive to *E.canis* (0.7 %), 2 cats to *E.ewingii* (0.3 %),1 cat to *E.chaffeensis* (0.2 %) and 4 cats were seroreactive with only the genus analyte. Seroreactivity to more than one FVBP was found in 16.1 % (9/56) of seroreactive cats. For each of these 9 cats, *B.burgdorferi* seroreactivity was detected in conjunction with either *A.phagocytophilum* (*n* = 7) or *E.ewingii* (*n* = 2) seroreactivity.

**Table 2:** Regional Seroprevalence by SNAP® M-A shown as percentages of 715 feline serum samples

Regions	*Anapl* genus	*Aph*	*Apl*	*Bb*	*Ehrl* genus	*Ec*	*Ech*	*Eew*
Northeast *n* = 187 (26 %)	2^a^	9 (6^a^)	0	26 (6^a^)	0	2	0	0
Mid Atlantic *n* = 59 (8 %)	2 (1^a^)	2	0	10 (3^a^)	1	0	0	2^a^
South *n* = 284 (40 %)	1	1	0	1	3	1	1	0
Midwest *n* = 114 (16 %)	0	1	0	1	1	0	0	0
West *n* = 42 (6 %)	0	0	0	0	0	1	0	0
Canada *n* = 26 (4 %)	0	0	0	0	0	1	0	0
Caribbean *n* = 3 (0.4 %)	0	0	0	1	0	0	0	0
Total *n* = 715	5 (0.7 %)	13 (1.8 %)	0	39 (5.5 %)	5 (0.7 %)	5 (0.7 %)	1 (0.2 %)	2 (0.3 %)

^a^indicates co-exposures

*Anapl* genus: *Anaplasma* genus*; Aph*: *A.phagocytophilum*; *Apl*: *A.platys*; *Bb*: *Borrelia burgdorferi*; *Ehrl* genus: *Ehrlichia* genus; *Ec*: *E.canis*; *Ech*: *E.chaffeensis*; *Eew*: *E.ewingii*

### PCR amplification of *Anaplasma* and *Ehrlichia*

Based upon conventional *Anaplasma* and *Ehrlichia 16S* rDNA PCR diagnostic results, performed at the time of sample submission to the VBDDL (*n* = 163), 7 cats were infected with either *A.phagocytophilum* (*n* = 4), *A.platys* (*n* = 2), or *E.canis* (*n* = 1). When archived frozen EDTA-whole blood (*n* = 331) was accessed for testing by *16S* rDNA PCR, 7 cats were infected with either an *Anaplasma* or *Ehrlichia* spp., including *E.ewingii* (*n* = 3), *E.chaffeensis* (*n* = 2), *A.phagocytophilum* (*n* = 1) and *A.platys* (*n* = 1). Based upon diagnostic and archival PCR testing, the total number of cats infected with an *Anaplasma* or *Ehrlichia* sp. was 3.2 % (13/406), with one *A.phagocytophilum* PCR positive cat represented in both the diagnostic and archival PCR results (Table 3). Additional retrospective *Anaplasma* and *Ehrlichia* genus- and species-specific PCRs confirmed several *16S* rDNA PCR positive samples, and included cats infected with *A.phagocytophilum*, *E.chaffeensis*, and *E.ewingii* (Table 3).

**Table 3:** State of origin, VBDDL diagnostic and archived sample Anaplasma and Ehrlichia PCR results, SNAP® M-A, SNAP® 4Dx®Plus and IFA (antigen) serology results for individual cats infected with FVBD

Cat #	US state of sample origin	VBDDL diagnostic PCR (gene targets)	PCR on archived samples (gene targets)	SNAP® M-A	SNAP® 4Dx® Plus	IFA (*Ech*)	IFA (*Ec*)	IFA (*Aph*)	IFA (*Bb*)
1	NC	*Apl (16S, GroEL, p44)*	N/A	(−)	N/A	N/A	N/A	N/A	N/A
2	OH	*Apl (16S)*	N/A	N/A	N/A	N/A	N/A	N/A	N/A
3	NC	ND	*Apl (16S)*	N/A	N/A	N/A	N/A	N/A	N/A
4	MI	*Aph (16S)*	(−)	N/A	N/A	N/A	N/A	N/A	N/A
5	MA	*Aph (16S)*	(−)	N/A	N/A	N/A	N/A	N/A	N/A
6	NC	*Aph (16S)*	(−)	N/A	N/A	N/A	N/A	N/A	N/A
7	NY	*Aph (16S)*	*Aph (16S, tr-1, p44)*	*Bb+*	*Bb+*	64	<16	<16	32
8	VA	*Ec (16S)*	(−)	N/A	N/A	N/A	N/A	N/A	N/A
9	NC	(−)	*Ech (16S)*	(−)	(−)	<16	<16	<16	128
10	MO	ND	*Ech (16S, sodB, nadA)*	(−)	(−)	<16	128	<16	16
11	SC	(−)	*Eew (16S)*	(−)	(−)	<16	<16	<16	16
12	CA	(−)	*Eew (16S)*	(−)	(−)	<16	<16	<16	<16
13	NC	(−)	*Eew (16S, sodB)*	N/A	N/A	N/A	N/A	N/A	N/A

*Aph*: *A.phagocytophilum*; *Apl*: *A.platys*; *Ec*: *E.canis*; *Ech*: *E.chaffeensis*; *Eew*: *E.ewingii*; *Bb*: *B.burgdorferi*; FVBD: feline vector-borne disease; ND: not done; N/A: Archived Serum or EDTA-whole blood was not available; (−): negative serology or PCR result

### Clinical data

Clinical data for 7 PCR positive cats is summarized in Table 4. The FVBP PCR positives for which records were available included 3 *A.platys* infected cats, one of which was a cat diagnosed with multiple myeloma, as previously described in a case report [22], 2 *A.phagocytophilum* infections, 1 *E.chaffeensis* infection (limited to CBC and biochemistry panel), and 1 *E.ewingii* infection. Four of the 6 cats were reported as being indoor/outdoor, 2 were indoor only, one unknown, and one cat had a tick removed immediately prior to becoming ill. Presenting complaints included anorexia, lethargy or epistaxis. Three cats were febrile at the time of physical examination and one cat was icteric, however this cat was co-infected with *Cytauxazoon felis* and *A.platys*. Feline Leukemia and Feline Immunodeficiency Virus (FeLV/FIV) results were negative for 6 of 6 cats tested. The most common hematological abnormalities included anemia (*n* = 5), thrombocytopenia (*n* = 5) and neutrophilia (*n* = 3). Other blood abnormalities reported by the attending veterinarian included hyperglobulinemia, lymphocytosis or thrombocytosis. One *E.ewingii* positive cat was found to be hyperthyroid.

**Table 4:** Abbreviated clinical data available for 7 Anaplasma or Ehrlichia PCR positive cats

Cat#	FVBD	Sig.	Presenting complaint	Abnormal PE findings	Hematological	Additional findings
1	*Apl*	11y MC DSH	chronic hyper-globulinemia	none reported	mod. hyperglobulinemia, mld. thrombocytopenia mld. anemia	indr/outdr; FeLV/FIV (−); co-infection with *Mh*, *Bh*, and *Bk*; dx. with multiple myeloma*
2	*Apl*	3y MC DSH	lethargy, pallor	pale, febrile (103.5 °C), heart murmur (IV/VI)	svr. anemia, mld leukocytosis mod. lymphocytosis	indr/outdr; FeLV/FIV (−); saline ag.(−); imaging: enlarged heart and mld. free fluid in thoracic and abdominal cavities; BM aspirate erythiroid hyperplasia and dysplasia, lymphoid hyperplasia; dx. PRCA or infection or CLL.
3	*Apl*	5y FS DMH	anorexia, lethargy, *C. felis* (+)	icteric, febrile (103.2 °C)	svr. anemia, mld. leukopenia mod. thrombocytopenia, mld. hyperproteinemia	indr/outdr; FeLV/FIV (−); normal coag-ulation (PT/PTT); co-infection *C. felis*
6	*Aph*	8 m MC DSH	lethargy, persistent leukocytosis	none reported	mod. neutrophilia, mod. lymphocytosis, mod. thrombocytosis	indr; FeLV/FIV (−); imaging WNL
7	*Aph*	9y MC DSH	lethargy, inappetance	lethargic, febrile (103.5 °C)	mld. anemia, mod. thrombocytopenia mod. neutrophilia mod. lymphopenia	indr/outdr; tick removed recently; FeLV/FIV (−); normal coagulation (PT/PTT)
9	*Ech*	11y MC DSH	ND	ND	WNL	ND
13	*Eew*	12y MC DSH	chronic intermittent epistaxis	epistaxis thyroid nodule	mod. anemia mld. neutrophilia svr. thrombocytopenia elevated T4	indr/outdr; FeLV/FIV (−); normal coagulation (PT/PTT); imaging WNL

*Aph*: *A.phagocytophilum*; *Apl*: *A.platys*; *Ech*: *E.chaffeensis*; *Eew*: *E.ewingii*; PE: physical examination; MC: male castrated; FS: female spayed; DS(M)H: domestic short (medium) hair; mld: mild; mod: moderate; svr: severe; indr: indoor; outdr: outdoor; WNL: within normal limits; ND: no data; FeLV: Feline Leukemia Virus; FIV: Feline Immunodeficiency Virus; BM: Bone Marrow; PRCA: pure red cell aplasia; CLL: Chronic Lymphoid Leukemia; PT: Prothrombin Time; PTT: Partial Thromboplastin Time. *previously reported case report [22]

### Comparison among serological assays

Fifty-nine SNAP® M-A positive serum samples had sufficient volume for SNAP®4Dx®Plus and/or IFA comparative testing. Of the 13 cats that were seroreactive to SNAP® M-A *Anaplasma* sp. analytes (EENZl and P44 Aph), only 3 (23 %) were seroreactive by SNAP®4Dx®Plus (EENZ1 analyte), whereas 11 (85 %) were IFA positive for *A.phagocytophilum* antibodies. When compared to the SNAP® M-A *Ehrlichia* sp. analytes (p30/p30-1, p16, VLPT, p28), there was variable agreement with the SNAP®4Dx®Plus and *E.chaffeensis* or *E.canis* IFA using whole cell antigen preparations (Table 5). By *E.chaffeensis* IFA, only one *E.chaffeensis* and one *Ehrlichia* genus reactive cat were seropositive at titers of 1:256 and 1:64, respectively. Only the *E.chaffeensis* IFA seroreactor was *E.canis* IFA seroreactive at a titer of 1:256. None of the five SNAP® M-A *E.canis* p16 reactors were positive by SNAP®4Dx®Plus (p16 not present in this assay) or by IFA testing using *E.chaffeensis* or *E.canis* whole cell antigens. Of the 39 SNAP® M-A *B.burgdorferi* C6 peptide positives, 23 (59 %) and 27 (69 %) were positive by SNAP®4Dx®Plus or a whole cell *B.burgdorferi* antigen preparation, respectively.

**Table 5:** Comparison of SNAP® M-A results for 59 sera with SNAP®4Dx®Plus and Anaplasma phagocytophilum, Ehrlichia chaffeensis, Ehrlichia canis, or Borrelia burgdorferi Immunofluorescence results

	# SNAP® M-A positive	# SNAP®4Dx®Plus positive	# IFA seroreactive ≥ 64	
SNAP® M-A Analyte			*A.phagocytophilum*	
*A.* genus eenz1	5	3/5	3/5	
*Aph* p44 aph	12	3/12	9/12	
*Apl* p44 apl	0	0	0	
			*E.chaffeensis*	*E.canis*
*E.* genus p30/p30-1	5	4/5	2/5	1/5
*Ec* p16	5	0/5	0/5	0/5
*Ech* VLPT	1	1/1	1/1	1/1
*Eew* p28	2	1/2	1/2	0/2
			*B.burgdorferi*	
*Bb* C6	39	23/39	27/39	

IFA: immunofluorescent assay; *A.*genus*: Anaplasma spp.; E.*genus*: Ehrlichia spp.; Aph*: *A. phagocytophilum*; *Apl*: *A.platys*; *Ec*: *E.canis*; *Ech*: *E.chaffeensis*; *Eew*: *E.ewingii*; *Bb*: *B. burgdorferi*

Serum samples, available for 4 PCR positive cats, (2 *E.chaffeensis* and 2 *E.ewingii*) were not seroreactive by any of the three serological assays with the exception of an antibody response of 1:128 against *E.canis* by IFA for a cat that was PCR positive for *E.chaffeensis* (Table 3). A serum sample available from a fifth cat PCR positive for *A.phagocytophilum* was reactive to the C6 *B.burgdorferi* analyte in both SNAP® M-A and SNAP®4Dx®Plus but was not reactive to any *A.phagocytophilum* antigens.

## Discussion

To the authors’ knowledge, this is one of the largest feline tick-borne pathogen prevalence studies reported for cats suspected of vector-borne infections from North America. Using species-specific peptides or PCR testing, this study identified two tick-borne pathogen species (*E.chaffeensis* and *E.ewingii)* that to our knowledge have not been previously reported to infect cats in North America. In addition, antibodies to *B.burgdorferi* (5.5 %) and *A.phagocytophilum* (1.8 %) were found frequently in serum submitted from sick cats in Lyme disease endemic regions of the northeastern and mid-Atlantic United States. These two pathogens share a common vector, *Ixodes scapularis*. Similarly, *B.burgdorferi* and *A.phagocytophilum* co-exposures were the most frequently detected. While exposure to, or infection with, a spectrum of FVBP were identified among these cats, the overall serological and PCR prevalence of tick-borne infections were relatively low compared to dogs tested during a similar time frame [26, 38]. For example, using SNAP® M-A to test over 6500 dog serum samples submitted to the NCSU-CVM-VBDDL from the United States, Canada and the Caribbean, primarily between 2008 and 2010, the overall canine seroprevalence was 8.3 % for *B.burgdorferi* and 3.4 % for *A.phagocytophilum,* both nearly double the prevalences in cats [26]. Reasonable explanations for the lower FVBP seroprevalences include shorter tick-attachment times due to fastidious grooming, thus reducing the opportunity for pathogen transmission. Also, although demographic and environmental data was not available for these diagnostic accessions, the cats included in this study were likely client-owned and therefore were more likely to be maintained primarily indoors and thus exposed to fewer ticks than their canine counterparts.

Historical or more recent documentation of exposure to, or infections with, *A.platys, E.canis, E.chaffeensis and E.ewingii* in cats provides a justification for future studies that investigate specific disease presentations associated with each of these infections. Similar to dogs, cats can be sequentially or concurrently exposed to more than one FVBP; therefore, co-infections can influence clinical, hematological and pathological findings [26]. In this study, complete or partial medical data obtained for 7 PCR positive cats (Table 4) underscores potential clinical and hematological disease similarities among cats and dogs in association with vector-borne infectious diseases. For example, four of seven cats were anemic and thrombocytopenic and epistaxis was reported in one of the markedly thrombocytopenic *E.ewingii* infected cats. Also, two *A.platys* infected cats were thrombocytopenic; however, co-infections were detected in both cats, so it is impossible to know the degree to which the *A.platys* infection contributed to thrombocytopenia in either cat.

In conjunction with published and ongoing studies of cat populations throughout the world, expanded FVBP test offerings by diagnostic laboratories are warranted. The sensitivity of a serological test is contingent upon the type, configuration and specificity of peptides chosen in the design of the assay. In addition, it should be determined if cats’ immunological reactivity to currently used diagnostic peptides is the same or different than dogs. Although assays being used in canine VBD diagnostic panels, whether ELISA or IFA based, will be a first step in facilitating the detection of FVBP in cats, improvements are in order. As an example, five cats were seroreactive using the *E.canis* p16 peptide, whereas none of these 5 cats were seroreactive by SNAP®4Dx®Plus (p16 not present in this assay) or by IFA testing using *E.chaffeensis* or *E.canis* whole cell antigens. Whether this discrepancy represents a lack of specificity of the p16 analyte, a lack of sensitivity of the commercial ELISA and IFA, or enhanced analytical sensitivity of this analyte for testing cat sera remains to be determined. Also, only one of the *E.chaffeensis* or *E.ewingii* infected cats (PCR+ with DNA sequence confirmation) was seroreactive, using any of the three assays assessed in this study. If as a general rule applicable to tick exposed cats, this finding could contribute to falsely low *Ehrlichia* seroprevalences, both diagnostically and during cat serosurvey studies. Isolation of FVBP from cats in conjunction with the detailed characterization of immunologic response to specific antigens may lead to assays that are more specific and hopefully more sensitive in the clinical diagnosis of acute or chronic vector-borne diseases in cats. The combination of serology and PCR testing has been recommended for the evaluation of canine VBDs [39]. Based upon the results of this study, using panels combining serology, with IFA being demonstrated as slightly more sensitive than ELISA in this study for *A.phagocytophilum, Ehrlichia spp.,* and *B.burgdorferi*, along with genus and species-specific PCR in cats seems warranted, as this approach would facilitate more accurate diagnoses and targeted therapy for sick cats.

There were several inherent limitations to this study. The cat serum and blood specimens submitted by veterinarians to the VBDDL were from cats presumably suspected of an infection with a FVBP. However, based upon the historical research priorities of our laboratory, it is likely that many of these specimens were submitted from cats in which cytauxzoonosis (*C. felis* transmitted by ticks) or bartonellosis (*Bartonella henselae* and other *Bartonella sp.* most often transmitted by fleas) were suspected. Thus, seroprevalence rates in these cats are presumably higher than in the healthy, client-owned cat population. Although cats were regionalized based on local veterinary hospitals or owner zip codes, individual travel histories were not available; therefore, where exposures or infections occurred remains uncertain. Also, as medical histories were not provided in conjunction with sample submission information, it was not possible to determine risk factors such as outdoor exposure potential (living primarily indoors, indoors and outdoors, or outdoors only), vector exposure and other environmental factors that would influence the prevalence results. Despite obtaining clinical data for 7 PCR positive cats; incomplete medical record entries, variability in the clinical data obtained for each cat and the documentation of co-infections in two cats, does not allow specific clinical or hematological abnormalities to be attributed to infection with a specific FVBP.

## Conclusions

Cats are exposed to and can be infected with vector-borne pathogens that commonly infect dogs and humans. *Anaplasma phagocytophilum, A.platys*, *E.canis*, *E.chaffeensis* and *E.ewingii* infections were confirmed by PCR amplification and DNA sequencing. To our knowledge, this study provides the first evidence for *E.chaffeensis* and *E.ewingii* infection in naturally-exposed cats in North America. Results from this study support the need for regional, serological and molecular FVBP prevalence studies, the need to further optimize serodiagnostic and PCR testing for cats, and the need for prospective studies to better characterize clinicopathological disease manifestations in cats infected with FVBP.

## Abbreviations

FVBP: Feline vector-borne pathogens
VBDDL: Vector-Borne Disease Diagnostic Laboratory
NCSU-CVM: North Carolina State University-College of Veterinary Medicine
SNAP® M-A: SNAP® Multi-Analyte Assay
qPCR: quantitative PCR
ELISA: Enzyme-linked immunosorbent assay
EDTA: Ethylenediaminetetraacetic acid
IFA: Immunofluorescent antibody
CBC: Complete blood count
FeLV/FIV: Feline leukemia / Feline immunodeficiency virus
PBS: Phosphate buffered saline

## References

[1] Levy JK, Lappin MR, Glaser AL, Birkenheuer AJ, Anderson TC, Edinboro CH (2011). Prevalence of infectious diseases in cats and dogs rescued following Hurricane Katrina. J Am Vet Med Assoc.

[2] Birkenheuer AJ, Le JA, Valenzisi AM, Tucker MD, Levy MG, Breitschwerdt EB (2006). *Cytauxzoon felis* in cats in the mid-Atlantic states: 34 cases (1998–2004). J Am Vet Med Assoc.

[3] Little SE (2010). Ehrlichiosis and anaplasmosis in dogs and cats. Vet Clin North Am Small Anim Pract.

[4] Lappin MR, Breitschwerdt EB, Jensen WA, Dunnigan B, Rha JY, Williams CR (2004). Molecular and serologic evidence of *Anaplasma phagocytophilum* infection in cats in North America. J Am Vet Med Assoc.

[5] Magnarelli LA, Bushmich SL, Ijdo JW, Fikrig E (2005). Seroprevalence of antibodies against *Borrelia burgdorferi* and *Anaplasma phagocytophilum* in cats. Am J Vet Res.

[6] Billeter SA, Spencer JA, Griffin B, Dykstra CC, Blagburn BL (2007). Prevalence of *Anaplasma phagocytophilum* in domestic felines in the United States. Vet Parasiol.

[7] Ayllón T, Diniz PPVP, Breitschwerdt EB, Villaescusa A, Rodríguez-Franco F, Sainz A (2012). Vector-borne diseases in client-owned and stray cats from Madrid, Spain. Vector Borne Zoo Dis.

[8] Braga Mdo S, André MR, Freschi CR, Teixeira MC, Machado RZ (2012). Molecular and serological detection of *Ehrlichia* spp. in cats on São Luís Island, Maranhão, Brazil. Rev Bras Parasitol Vet.

[9] Braga IA, Santos LG, Melo AL, Jaune FW, Ziliani TF, Girardi AF (2013). Hematological values associated to the serological and molecular diagnostic in cats suspected of *Ehrlichia canis* infection. Rev Bras Parasitol Vet..

[10] Beaufils JP, Marin-Granel J, Jumelle P (1995). *Ehrlichia* infection in cats: a review of three cases. Prat Med Chir Anim Comp.

[11] Maia C, Ramos C, Coimbra M, Bastos F, Martins A, Pinto P (2014). Bacterial and protozoal agents of feline vector-borne diseases in domestic and stray cats from southern Portugal. Parasit Vectors.

[12] Spada E, Proverbio D, Galluzzo P, Della Pepa A, Perego R, Bagnaqatti De Giorgi GB (2014). Molecular study on selected vector-borne infections in urban stray colony cats in northern Italy. J Fel Med Surg.

[13] Aguirre E, Tesouro MA, Amusategui I, Rodriguez-Franco F, Sainz A (2004). Assessment of feline ehrlichiosis in central Spain using serology and a polymerase chain reaction technique. Ann N Y Acad Sci.

[14] Ortuno A, Gauss CBL, Garcia F, Gutierrez JF (2005). Serological evidence of *Ehrlichia* spp. exposure in cats from Northeastern Spain. J Vet Med.

[15] Bjöersdorff A, Svendenius L, Owens JH, Massung RF (1999). Feline granulocytic ehrlichiosis—a report of a new clinical entity and characterization of the infectious agent. J Small Animal Practice.

[16] Matthewman LA, Kelly PJ, Wray K, Bryson NR, Rycroft AN, Raoult D (1996). Antibodies in cat sera from southern Africa react with antigens of *Ehrlichia canis*. Vet Rec.

[17] Maruyama S, Sakai T, Morita Y, Tanaka S, Kabeya H, Boonmar S (2001). Prevalence of *Bartonella* species and 16 s rRNA gene types of *Bartonella henselae* from domestic cats in Thailand. Am J Trop Med Hyg.

[18] Yin-Chachun, Liu-Hungjen, Lin-Suenchuain, Liao-Minghuei, Wu-Yeonghuey (2003). Identification of *Ehrlichia canis* in cats by nested polymerase chain reaction and nucleotide sequence analysis. Taiwan Vet J.

[19] Solano-Gallego L, Hegarty B, Espada Y, Llull J, Breitschwerdt E (2006). Serological and molecular evidence of exposure to arthropod-borne organisms in cats from northeastern Spain. Vet Microbiol.

[20] Ebani VV, Bertelloni F (2014). Serological evidence of exposure to *Ehrlichia canis* and *Anaplasma phagocytophilum* in Central Italian healthy domestic cats. Ticks Tick Borne Dis.

[21] Lima MLF, Soares PT, Ramos CAN, Araújo FR, Ramos RAN, Souza IIF (2010). Molecular detection of *Anaplasma platys* in a naturally-infected cat in Brazil. Brazilian J Microbiol.

[22] Qurollo BA, Balakrishnan N, Cannon CZ, Maggi RG, Breitschwerdt EB (2014). Co-infection with *Anaplasma platys, Bartonella henselae, Bartonella koehlerae* and ‘*Candidatus* Mycoplasma haemominutum’ in a cat diagnosed with splenic plasmacytosis and multiple myeloma. J Feline MedSurg.

[23] Breitschwerdt EB, Hancock SI, Hegarty BC, Martin-Granel J, Jumelle P, Barbault-Jumelle M (2002). Ehrlichiose feline: identification genetique de l’agent chez deux chats. Prat Med Chir Anim Comp.

[24] Breitschwerdt EB, Abrams-Ogg AC, Lappin MR, Bienzle D, Hancock SI, Cowan SM (2002). Molecular evidence supporting *Ehrlichia canis*-like infection in cats. J Vet Intern Med.

[25] de Oliveira LS, Moura LC, Oliveira KA, Agostini M, de Oliveira AC, de Almeida MR (2009). Molecular detection of *Ehrlichia canis* in cats in Brazil. Clin Microbiol Infect.

[26] Qurollo BA, Chandrashekar R, Hegarty BC, Beall MJ, Stillman B, Liu J (2014). Vector-borne pathogen exposure in dogs in North America and the Caribbean as assessed by *Borrelia burgdorferi, Anaplasma* and *Ehrlichia* species-specific peptides. Infect Ecol Epidemiol.

[27] Starkey LA, Barrett AW, Chandrashekar R, Stillman BA, Tyrrell P, Thatcher B (2014). Development of antibodies to and PCR detection of *Ehrlichia* spp. in dogs following natural tick exposure. Vet Microbiol.

[28] Levy SA, O’Connor TP, Hanscom JL, Shields P (2003). Evaluation of a canine C6 ELISA lyme disease test for the determination of the infection status of cats naturally exposed to *Borrelia burgdorferi*. Vet Therapeutics.

[29] Chandrashekar R, Daniluk D, Moffitt S, Lorentzen L, Williams J (2008). Serologic diagnosis of equine borreliosis: evaluation of an in-clinic enzyme-linked immunosorbent assay (SNAP 4Dx). Intern J Appl Res Vet Med.

[30] Duell JR, Carmichael R, Herrin BH, Holbrook TC, Talley J, Little SE (2013). Prevalence and species of ticks on horses in central Oklahoma. J Med Entomol.

[31] O’Nion VL, Montilla HJ, Qurollo BA, Maggi RG, Hegarty BC, Tornquist J (2015). Potentially novel *Ehrlichia* species in horses, Nicaragua. Emerg Infect Dis.

[32] Dahlgren FS, Mandel EJ, Krebs JW, Massung RF, McQuiston JH (2011). Increasing incidence of *Ehrlichia chaffeensis* and *Anaplasma phagocytophilum* in the United States, 2000–2007. Am J Trop Med Hyg.

[33] McQuiston JH, McCall CL, William L, Nicholson WL (2003). Ehrlichiosis and related infections. J Am Vet Med Assoc.

[34] Thomas RJ, Dumler JS, Carlyon JA (2009). Current management of human granulocytic anaplasmosis, human monocytic ehrlichiosis and *Ehrlichia ewingii* ehrlichiosis. Expert Rev Anti Infect Ther.

[35] Eddlestone SM, Diniz PPVP, Neer TM, Gaunt SD, Corstvet R, Cho D (2007). Doxycycline clearance of experimentally induced chronic *Ehrlichia canis* infection in dogs. J Vet Intern Med.

[36] Qurollo BA, Riggins D, Comyn A, Zewde MT, Breitschwerdt EB (2014). Development and validation of a sensitive and specific *sodB*-based quantitative PCR assay for molecular diagnosis of *Ehrlichia* species. J Clin Microbiol.

[37] Breitschwerdt EB, Hegarty BC, Hancock SI (1998). Sequential evaluation of dogs naturally infected with *Ehrlichia canis*, *Ehrlichia chaffeensis*, *Ehrlichia equi*, *Ehrlichia ewingii*, or *Bartonella vinsonii*. J Clin Microbiol.

[38] Yancey CB, Hegarty BC, Qurollo BA, Levy MG, Birkenheuer AJ, Weber DJ (2014). Regional seroreactivity and vector-borne disease co-exposures in dogs in the United States from 2004–2010: utility of canine surveillance. Vector Borne Zoonotic Dis.

[39] Maggi RG, Birkenheuer AJ, Hegarty BC, Bradley JM, Levy MG, Breitschwerdt EB (2014). Advantages and limitations of serological and molecular panels for the diagnosis of vector-borne infectious diseases in dogs. Parasit Vectors.

